# Identification and characterisation of eight novel *SERPINA1* Null mutations

**DOI:** 10.1186/s13023-014-0172-y

**Published:** 2014-11-26

**Authors:** Ilaria Ferrarotti, Tomás P Carroll, Stefania Ottaviani, Anna M Fra, Geraldine O’Brien, Kevin Molloy, Luciano Corda, Daniela Medicina, David R Curran, Noel G McElvaney, Maurizio Luisetti

**Affiliations:** Centre for Diagnosis of Inherited Alpha-1 Antitrypsin Deficiency, Laboratory of Biochemistry and Genetics, Institute for Respiratory Disease, Fondazione IRCCS Policlinico San Matteo, Pavia, Italy; Respiratory Research, Department of Medicine, Royal College of Surgeons in Ireland Education and Research Centre, Beaumont Hospital, Dublin, Ireland; Department of Molecular and Translational Medicine, University of Brescia, Brescia, Italy; Department of Internal Medicine, Respiratory Disease Unit, Spedali Civili, Brescia, Italy; Department of Pathology, Spedali Civili of Brescia, Brescia, Italy; Respiratory Department, Mercy University Hospital, Cork, Ireland; Department of Molecular Medicine, University of Pavia, Pavia, Italy

**Keywords:** Alpha-1 antitrypsin deficiency, Q0 mutation, Lung diseases, Serpins

## Abstract

**Background:**

Alpha-1 antitrypsin (AAT) is the most abundant circulating antiprotease and is a member of the serine protease inhibitor (SERPIN) superfamily. The gene encoding AAT is the highly polymorphic *SERPINA1* gene, found at 14q32.1. Mutations in the *SERPINA1* gene can lead to AAT deficiency (AATD) which is associated with a substantially increased risk of lung and liver disease. The most common pathogenic AAT variant is Z (Glu342Lys) which causes AAT to misfold and polymerise within hepatocytes and other AAT-producing cells. A group of rare mutations causing AATD, termed Null or Q0, are characterised by a complete absence of AAT in the plasma. While ultra rare, these mutations confer a particularly high risk of emphysema.

**Methods:**

We performed the determination of AAT serum levels by a rate immune nephelometric method or by immune turbidimetry. The phenotype was determined by isoelectric focusing analysis on agarose gel with specific immunological detection. DNA was isolated from whole peripheral blood or dried blood spot (DBS) samples using a commercial extraction kit. The new mutations were identified by sequencing all coding exons (II-V) of the *SERPINA1* gene.

**Results:**

We have found eight previously unidentified *SERPINA1* Null mutations, named: Q0_cork_, Q0_perugia_, Q0_brescia_, Q0_torino_, Q0_cosenza_, Q0_pordenone_, Q0_lampedusa_, and Q0_dublin_ . Analysis of clinical characteristics revealed evidence of the recurrence of lung symptoms (dyspnoea, cough) and lung diseases (emphysema, asthma, chronic bronchitis) in M/Null subjects, over 45 years-old, irrespective of smoking.

**Conclusions:**

We have added eight more mutations to the list of *SERPINA1* Null alleles. This study underlines that the laboratory diagnosis of AATD is not just a matter of degree, because the precise determination of the deficiency and Null alleles carried by an AATD individual may help to evaluate the risk for the lung disease.

**Electronic supplementary material:**

The online version of this article (doi:10.1186/s13023-014-0172-y) contains supplementary material, which is available to authorized users.

## Background

Alpha-1 antitrypsin (AAT) is a serine protease inhibitor, encoded by the *SERPINA1* gene on the long arm of chromosome 14 at 14q32.1. The gene is comprised of four coding exons (II, III, IV, and V), three untranslated exons (Ia, Ib, and Ic) in the 5′ region and six introns. Following translation, the 24 amino acid signal peptide is removed and the mature polypeptide is a 394 amino acid, 52 kDa glycoprotein with three asparagine-linked carbohydrate side chains [1]. AAT is an acute phase protein produced predominantly by hepatocytes, but AAT synthesis also occurs in mononuclear phagocytes, neutrophils, and airway and intestinal epithelial cells [2]. Consistent with a role as an important acute phase reactant, hepatocytes express approximately 200 times more AAT mRNA than other cells [3] and serum levels rapidly increase several-fold during the acute phase response [4]. The primary function of AAT is the regulation of serine proteases, and the chief site of action is the lungs where it protects the fragile alveolar tissues from proteolytic degradation during inflammatory responses. In addition to its undoubted anti-protease properties, there is accumulating evidence that AAT plays a key anti-inflammatory role [5].

Alpha-1 antitrypsin deficiency (AATD) (MIM # 613490) is an inherited condition caused by mutations within the polymorphic *SERPINA1* gene and is characterised by decreased serum AAT concentrations. AATD is an under-diagnosed condition and the majority of cases remain undiagnosed. The World Health Organisation (WHO), the American Thoracic Society (ATS), and the European Respiratory Society (ERS) advocate a targeted screening approach for the detection of AATD in at risk populations, specifically chronic obstructive pulmonary disease (COPD), non-responsive asthma, cryptogenic liver disease and in first degree relatives of known AATD patients. Over 100 mutations leading to AAT deficiency have been identified to date and are associated with varying degrees of risk for lung and liver disease. AATD is associated with increased risk of cutaneous panniculitis [6] and case reports have linked AATD to vasculitis [7], and Wegener’s granulomatosis [8] with the Z allele over-represented in subsets of ANCA-associated vasculitis [9]. The most common mutations known to cause AATD are the dysfunctional Z (Glu342Lys) and S (Glu264Val) mutations. The Z mutation leads to a severe plasma deficiency and is the most common clinically significant allele. The majority of individuals diagnosed with severe AATD are homozygous for the Z mutation, and have circulating AAT levels reduced to 10-15% of normal. This is because the Z mutation prompts the AAT protein to polymerise and accumulate within the endoplasmic reticulum of hepatocytes, thus causing impaired secretion [10]. The rate of polymer formation for S is much slower than Z AAT, leading to reduced retention of protein within hepatocytes, milder plasma deficiency, and a negligible risk of disease in MS heterozygotes [11,12]. However, there is a risk of lung disease in compound heterozygotes. For example, if the slowly polymerising S variant of AAT is inherited with a rapidly polymerising variant such as Z, the two variants when co-expressed can interact to form heteropolymers, leading to cirrhosis and plasma deficiency [13].

The ultra rare family of *SERPINA1* mutations termed silent or Null are characterised by a complete absence of AAT in the plasma. Null (also called Q0) mutations are caused by a variety of different mechanisms including large gene deletions [14], intron mutations [15], nonsense mutations [16], and frameshift mutations [17]. In some cases, Null variants are synthesised in the hepatocytes, but they are rapidly cleared by intracellular degradation pathways [18]. As Null mutations do not induce AAT polymerisation, they confer no risk of liver disease but do confer a particularly high risk of lung disease [19]. The exact prevalence of Null mutations is unclear, and is hampered by a lack of general awareness of AATD and inherent flaws in diagnostic strategies.

We report here eight cases of previously unidentified Null *SERPINA1* mutations in the Italian and Irish populations.

## Methods

The diagnostic algorithm for diagnosis of AATD was applied as previously reported [20]. The probands were referred to the Italian or Irish National Reference Centres for the Diagnosis of AATD, situated in Pavia and Brescia (Italy), and Dublin (Ireland), respectively. Where possible, relatives were analysed and family trees were created (online Additional file 1). Family members included in the study or their parents gave written informed consent. All procedures were in accordance with the declaration of Helsinki and approved by the local ethics committees. Clinical data were obtained from direct observation or medical charts.

AAT measurements were performed by a rate immune nephelometric method (Array 360 System; Beckman-Coulter) or by immune turbidimetry (Beckman Coulter AU5400). The phenotype was determined by isoelectric focusing analysis (IEF) on agarose gel with specific immunological detection [21]. DNA was isolated from whole peripheral blood or dried blood spot (DBS) samples using a commercial extraction kit (DNA IQ System, Promega or PAXgene Blood DNA kit, PreAnalytix or DNA Blood Mini kit, Qiagen). The new mutations were identified by sequencing all coding exons (II-V) of the AAT gene (*SERPINA1*, RefSeq: NG_008290), as previously described [20,22], using the CEQ 8800 genetic analysis System (Beckman Coulter) or the Big Dye Terminator Cycle Sequencing Kit 3.1 (Applied Biosystem) with the 3130 Genetic Analyzer.

## Results

**Figure 1:**
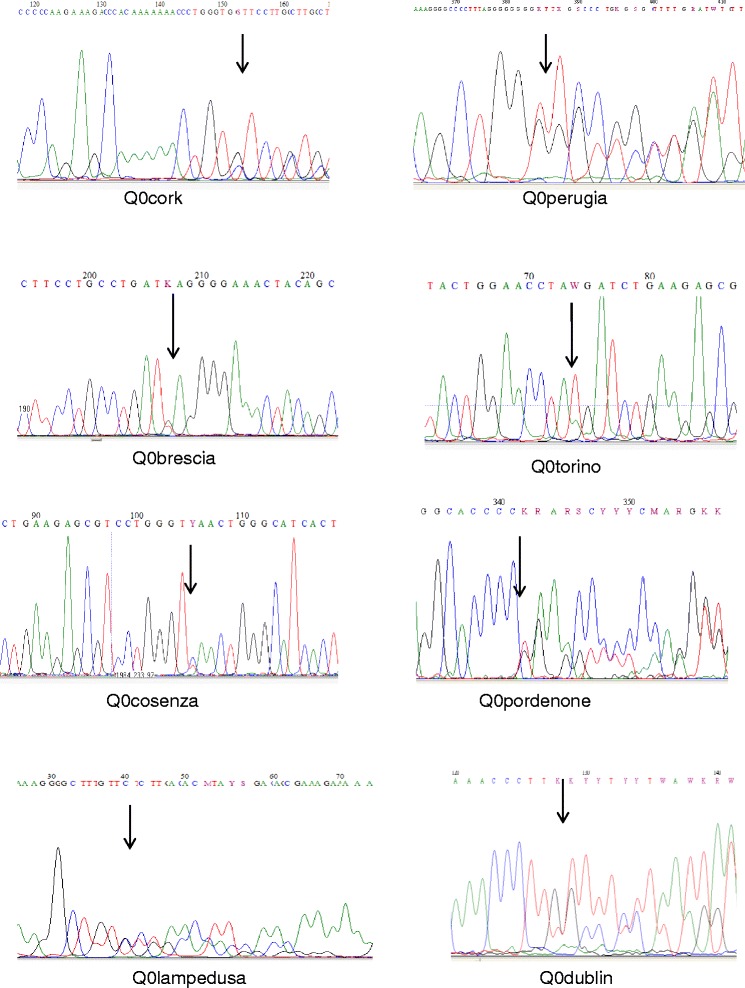
**Genomic sequence chromatograms representing** ***SERPINA1*** **Null mutations.**

The specific mutations are summarized in Table 1. The eight new Null mutations have been conventionally named Q0_cork_, Q0_perugia_, Q0_brescia_, Q0_torino_, Q0_cosenza_, Q0_pordenone_, Q0_lampedusa_, and Q0_dublin_ according to the birthplaces of the oldest subject carrying each mutation. Q0_brescia_, Q0_torino_ and Q0_cosenza_ consist of point mutations in the sequence of coding DNA that result in a premature stop codon (nonsense mutation). Q0_cork_, Q0_perugia_, Q0_pordenone_, Q0_lampedusa_ and Q0_dublin_ were caused by deletions, resulting in frameshift of the reading frame, and creating premature stop codons (Figure 1).

**Table 1:** **Description of the eight new** ***SERPINA1*** **Null mutations identified**

**Variant**	**Mutation**
**Q0cork**	T180ACA,delCA > Ter190TAA
**Q0perugia**	V239GTG, delG > Ter241TGA
**Q0brescia**	E257GAG > TerTAG
**Q0torino**	Y297TAT > TerTAA
**Q0cosenza**	Q305CAA > TerTAA
**Q0pordenone**	L327CTG,delT > Ter338TGA
**Q0lampedusa**	V337GTG,delG > Ter338TGA
**Q0dublin**	F370TTT,delT > Ter373TAA

The genotype, AAT levels, and clinical details of each proband and their relatives bearing Null mutations are listed in Table 2.

**Table 2:** **Summary of clinical details of Q0 individuals**

**Case**	**Genotype**	**AAT (g/L)**	**Age at diagnosis (years)**	**Proband/kinship to proband**	**Clinical**	**Pack/year**
**1.1 – IA**	M1/Q0cork	0.70	43	proband	cough, dyspnoea	15
**2.1 - IA**	M3/Q0perugia	0.83	60	proband	emphysema	52
2.1 - IB	M3/Q0perugia	0.80	54	brother	healthy	22
3.1 – IA	M1/Q0brescia	0.70	73	mother	chronic bronchitis	0
3.1 – IB	M1/Q0brescia	0.71	71	father	healthy	0
**3.1 – IIA**	Q0brescia/Q0brescia	Undetectable	41	proband	emphysema	3
**3.1 – IIB**	Q0brescia/Q0brescia	Undetectable	37	proband	emphysema	6
3.1 – IIIA	M1/Q0brescia	0.70	7	daughter	healthy	0
3.1 – IIIB	M1/Q0brescia	1.08	10	daughter	healthy	0
**3.2 - IA**	Z/Q0brescia	0.20	41	proband	emphysema	20
**4.1 - IA**	S/Q0torino	0.71	53	proband	emphysema	15
5.1 – IA	M2/Q0cosenza	0.80	63	aunt	dyspnoea	0
5.1 – IB	M2/Q0cosenza	0.85	67	mother	asthma	0
**5.1 – IIA**	S/Q0cosenza	0.60	34	proband	healthy	4
5.1 – IIB	S/Q0cosenza	0.71	43	sister	healthy	0
6.1 – IA	M2/Q0pordenone	0.55	37	father	healthy	0
6.1 – IIA	M3/Q0pordenone	0.81	6	brother	healthy	0
**6.1 – IIB**	M1/Q0pordenone	0.77	0.5	proband	cough	0
**6.2 – IA**	M1/Q0pordenone	0.91	54	proband	emphysema	ex
6.2 – IIA	M1/Q0pordenone	0.78	34	nephew	healthy	0
6.2 – IIB	M1/Q0pordenone	0.66	24	nephew	healthy	0
**6.3 – IA**	M1/Q0pordenone	0.47	69	proband	emphysema	unknown
6.3 – IIA	M1/Q0pordenone	0.81	37	son	healthy	0.1
6.4 – IA	M3/Q0pordenone	1.30	38	mother	healthy (pregnant)	0
**6.4 – IIA**	M2/Q0pordenone	0.78	6	proband	healthy	0
7.1 – IA	M1/Q0lampedusa	0.62	87	mother	emphysema	0
7.1 – IIA	M1/Q0lampedusa	0.76	57	sister	healthy	0
7.1 – IIB	M1/Q0lampedusa	0.80	61	sister	healthy	0
7.1 – IIC	M1/Q0lampedusa	0.73	55	sister	healthy	15
7.1 – IID	M1/Q0lampedusa	0.74	51	sister	chronic bronchitis	60
7.1 – IIE	M1/Q0lampedusa	0.71	48	sister	chronic bronchitis	0
**7.1 – IIF**	Q0lampedusa/Q0lampedusa	<0.1	46	proband	emphysema, asthma	0
7.1 – IIIA	M1/Q0lampedusa	1.00	30	niece	healthy	0
7.1 – IIIB	M2/Q0lampedusa	0.84	39	nephew	healthy	10
7.1 – IIIC	M1/Q0lampedusa	0.64	33	nephew	healthy	24
7.1– IIID	M1/Q0lampedusa	0.74	22	nephew	healthy	1.5
7.1 – IIIE	M1/Q0lampedusa	0.67	15	nephew	healthy	0
**8.1 – IA**	M1/Q0dublin	0.74	70	proband	bronchiectasis	1
8.1 – IIA	M1/Q0dublin	0.64	40	son	healthy	0
8.1 – IIB	M1/Q0dublin	0.70	34	son	recurrent LRTIs	1
8.1 – IIC	M1/Q0dublin	1.11	39	daughter	healthy	0

Index cases are written in bold.

### Q0_cork_

The proband was a 43 year old female who presented with cough, dyspnoea and wheeze, and was subsequently diagnosed with asthma by methacholine challenge test (Family 1.1 – subject IA, Table 2). A current smoker, spirometry showed no evidence of airways obstruction with pre-bronchodilator FEV1 of 2.55 L (95%), FVC 3.12 L (100%), and FEV1/FVC 82%. High resolution computed tomography (HRCT) of the lungs showed no evidence of emphysema or bronchiectasis. However, during routine assessment, the AAT concentration was found to be unusually low given the apparent MM phenotype observed on IEF, therefore DNA sequencing was performed. A deletion of CA in codon 180 ACA (exon II), present in heterozygosity, was detected. The deletion causes a frameshift in the reading frame and generates a premature stop codon (TAA) downstream at codon 190. The subject was homozygous Val213, therefore the novel Q0_cork_ deletion arose on a M1(Val213) background.

### Q0_perugia_

The proband (Family 2.1 – subject IA, Table 2) was a 60 year old male heavy smoker who developed emphysema before the age of 50. Since his AAT concentration in plasma was lower than normal, a complete genetic analysis of AAT was performed. The sequencing of *SERPINA1* gene revealed the heterozygous deletion of the first G in the codon 239 GTG (exon III), which causes a frameshift in the reading frame and the generation of a premature stop codon (241TGA). The novel mutation was also detected in a brother. Phenotype analysis and family pedigree revealed that this Null mutation arose on M1(Val213) background.

### Q0_brescia_

The probands were two sisters (Family 3.1, Table 2), both suffering from pulmonary emphysema and COPD. Their spirometry values showed obstructive defects with pre-bronchodilator FEV1 of 1.89 and 1.23 L (62% and 38%), FVC 3.38 and 2.11 L (97% and 63%), and FEV1/FVC 64% and 60%, respectively. Direct sequencing revealed both are homozygous for a point mutation at codon 257 (G > T transversion), changing a GAG (glutamic acid) codon into a TAG stop codon. Furthermore, both were homozygous for Alanine polymorphism at position 213 (rs6647), corresponding to the ancestral AAT gene variant M1(Ala). The familial study was performed on their two daughters (one from each sister) and their parents and confirmed the mendelian inheritance, showing heterozygosity both for the mutation at position 257 and for the M1 polymorphism at position 213 for all subjects. According to the reports of the probands their parents had no distant relationship, although they were born in two nearby villages in south-east Italy. Subsequently, this novel mutation was detected in a patient with severe AATD who was found to be composite heterozygous Z/Q0_brescia_ (3.2-IA, Table 2). The proband, born in the same south-eastern Italian area, was a heavy smoker, who suffered from dyspnoea on exertion and productive cough, and he developed panlobular emphysema by the age of 40. His spirometry values showed obstructive defects with pre-bronchodilator FEV1 of 1.01 L (27%), FVC 3.36 L (73%), and FEV1/FVC 36%.

### Q0_torino_

In index case 4.1 – IA (Table 2) DNA sequencing revealed heterozygosity for the S mutation (rs17580) and for a T > A transversion at codon 297 (TyrTAT > TerTAA) in exon IV. Analysis of the daughter confirmed that the Null mutation does not segregate with the S mutation and it arose on a M1(Val) background. The proband was a ex-smoker (15 pack/year) with emphysema and dyspnoea at rest.

### Q0_cosenza_

The proband was a 34 year old healthy male with a reported low concentration of AAT in plasma during a routine medical assessment (Family 5.1 – subject IIA, Table 2). DNA sequencing of the proband revealed heterozygosity for the S mutation (rs17580) and for a C > T transition at codon 305 (CAA > TAA) in exon IV. This transversion results in a premature Stop codon instead of a glutamine codon. Family screening revealed that the Null mutation does not segregate with S mutation and that the novel Q0_cosenza_ allele arose on an M2 background. The novel mutation was also detected in a sister (who carried the S mutation as well), the mother and an aunt.

### Q0_pordenone_

In index case 6.1 – IIB (Table 2), a deletion of a single T in codon 327 (exon IV) was discovered by DNA sequencing. The deletion was heterozygous and no other mutation was present. It causes a frameshift in the reading frame and generates a premature stop codon (TGA) 11 codons downstream. The mutation was also detected in the father and a brother of the index case. Like in the index case 6.1-IIB, Q0_pordenone_ was identified in heterozygosity with M alleles coding for normal AAT levels in 3 additional cases (6.2 - IA, 6.3 - IA and 6.4 - IIA), and in 4 relatives (2 nephews of 6.2 - IA, one son of 6.3 - IA and the mother of 6.4 - IIA). The four families carrying this novel Null allele were not related, but all subjects carrying Q0_pordenone_ identified so far were born in the North-East region of Italy.

### Q0_lampedusa_

DNA sequencing of the 4 exons of *SERPINA1* in the index case (7.1 – IIF, Table 2) revealed a homozygous deletion of a single G in codon 337 (exon V), occurring in the background of a normal M2 allele (His101-Val213-Asp376). This deletion results in a frameshift that produces an altered reading frame and generates an immediately adjacent premature stop codon (TGA) at position 338. The proband was a woman, never smoker, who worked in a sawmill; she had the first episodes of dyspnoea on exertion at the age of 35, but suspicion of AATD did not arise untill ten years later, when HRCT diagnosed centrolobular emphysema and spirometry detected slight obstruction with pre-bronchodilator FEV1 of 1.5 (63%), FVC 2.39 L (85% ), and post-bronchodilator FEV1 of 1.63 (72%), FVC 2.65 L (96%). The consanguinity of the proband’s parents was excluded, according to the direct report of the patients; nevertheless, they was born in two small islands near to Sicily, therefore a founder effect is probable. The novel mutation Q0_lampedusa_ was subsequently diagnosed in heterozygous fashion with M alleles coding for normal AAT levels in 11 out of 23 relatives who were subsequently investigated. Direct sequencing of *SERPINA1* exons in the remaining family members has confirmed the segregation of the mutant allele.

### Q0_dublin_

The proband was a 70 year old female who presented with bronchiectasis and a lower than expected AAT concentration given the apparent MM phenotype observed on IEF analysis (Family 8.1 – subject IC, Table 2). A past smoker, spirometry showed no evidence of airways obstruction with pre-bronchodilator FEV1 of 1.44 L (89%), FVC 1.97 L (98%), and FEV1/FVC 73%. HRCT of the lungs showed bronchiectasis but no evidence of emphysema. Sequencing identified a deletion of a single T resulting in a frameshift which alters the reading frame and generates an adjacent premature stop codon (TAA) at position 373. The subject was homozygous Val213, therefore the novel Q0_dublin_ deletion arose on a M1(Val213) background. The same mutation was detected in heterozygosity in all three children.

## Discussion

Null alleles result from different molecular mechanisms, including large gene deletions, intron mutations, nonsense mutations, frameshift mutations due to small insertions or deletions, and missense mutations associated with amino acid substitutions in potentially critical structural elements [23]. The common trait of Null mutations is the total absence of serum AAT. These mutations are extremely rare and can be difficult to diagnose, mainly because isoelectric focusing (IEF), a commonly used diagnostic method, although not preferred technique for screening of AATD [24], is not able to detect Null variants, as they do not produce protein. Therefore, the M/Null and MM phenotypes are identical when analysed by isoelectric focusing with only the normal M protein evident. Secondly, M/Null genotypes can be misclassified as M homozygotes in many common genotyping assays [25]. Sequence analysis of *SERPINA1* gene is the optimal technique to detect Null mutations and only the application of an efficient and cost-effective diagnostic algorithm can ensure the diagnosis of a subject heterozygous or homozygous for Null mutations [20].

The existence of AAT Null alleles was first noted in the early 1970s by several investigators. The first published report of a Null *SERPINA1* mutation described the case of a 24 year old man who had advanced pulmonary emphysema and no detectable serum AAT [26]. The first report of a probable Null *SERPINA1* mutation in Ireland was a case report in 1974 describing a pedigree in which the proband was Z/Null, a son S/Null and the mother M/Null [27]. The precise Null mutation was not identified and the diagnosis was based on the discordant AAT concentrations in his son and mother when compared to phenotype identified by starch gel electrophoresis. The first report of a Null mutation of Italian origin was Q0_trastevere_, which was detected in an Italian individual with asthma and emphysema [16].

To date, a total of 26 different Null alleles have been detected and characterized (Table 3). Many are caused by premature stop codons, mainly due to nonsense mutations or insertion/deletion of one-two nucleotides that cause frameshift of the reading frame and lead to a premature stop codon. A second group of Null mutations lie in introns; some of these have been identified in mRNA splicing sites: Null_west_ is characterized by a single G > T base substitution at position 1 of intron II, which generally is highly conserved; Null_bonny blue_ has been described as a deletion of the previously reported G. Other mutations are caused by large deletions; examples are Null_isola di procida_, a deletion of a 17Kb fragment that includes exons II-V [14], and Null_riedenburg_, caused by the complete deletion of the gene [28]. It is well known that an almost full length molecule is essential for the secretion of AAT, therefore a truncated protein prevents the secretion itself [18].

**Table 3:** **List of the 24 Null mutation** ***SERPINA1*** **described to date**

**Mechanism**	**Allele**	**Intron/exon**	**Mutation**	**Reference**
Large deletion	Q0isola di procida	Intron IC	g8801,del17.65 kb	[14]
	Q0riedenburg	Exon IC	Complete deletion of the gene	[28]
Intron mutations	Q0savannah	Intron IA	g.5307_5308ins8bp	[29]
	Q0porto	Intron IC	+1G > A	[30]
	Q0madrid	Intron IC	+3, insT	[31]
	Q0west	Intron II	+1G > T	[15]
	Q0bonny blue	Intron II	+1delG	[23]
Nonsense mutations	Q0kowloon	Exon II	Y 38TAC > Ter TAA	[23]
	Q0chillichote	Exon II	Q 156CAG > Ter TAG	[29]
	Q0amersfoort or Q0predevoort rs199422210	Exon II	Y 160TAC > Ter TAG	[19,32]
	Q0trastevere	Exon III	W194 TGG > Ter TGA	[16]
	Q0bellingham rs199422211	Exon III	K 217AAG > Ter TAG	[33]
	Q0cairo rs1802963	Exon III	K 259AAA > Ter TAA	[34]
Frameshift mutations	Q0milano	Exon III	K59,del17bp > Ter AAA	[35]
	Q0soest	Exon II	T102ACC,del A > Ter 112 TGA	[32]
	Q0granite falls rs267606950	Exon II	YTAC, delC > Ter 160 TAG	[36]
	Q0hong kong	Exon IV	L318CTC, del TC > Ter 334 TAA	[17]
	Q0mattawa rs28929473	Exon V	L353 TTA, ins T > Ter 376 TGA	[37]
	Q0ourem	Exon V	L 352TTA, ins T > Ter 376 TGA	[38]
	Q0bolton	Exon V	P362CCC, delC > Ter 373 TAA	[39]
	Q0clayton	Exon V	P 362CCC,ins C > Ter 376 TGA, and M1(Val)	[40]
	Q0saarbruecken	Exon V	P362CCC,ins C > Ter 376 TGA, and M1(Ala)	[41]
Missense mutations	Q0lisbon	Exon II	T68ACC > I ATC	[41]
	Q0ludwigshafen rs28931572	Exon II	I92ATC > N AAC	[42]
	Q0newport	Exon II	G115GGC > S AGC	[43]
	Q0new hope	Exon IV	G320GGG > E GAG and E342GAG > L AAG	[23]

For intronic mutations, reference sequence was NG_008290; dbSNP identification was reported where present.

Interestingly, Null mutations can also be induced by a simple amino acid substitution, like in Null_ludwigshafen_ (Ile^92^ > Asn^92^). This substitution of a polar for a non-polar amino acid leads to folding impairment, with destruction of tertiary structure and therefore intracellular degradation [42]. In most Null mutations belonging to this group, it is not clear whether the altered glycoprotein is unstable and therefore recognized as defective by intracellular methabolic pathways and degraded, or if it is secreted but, due to a very short half-life with rapid turnover, it cannot be detected by routine diagnostic assays. In addition, some Null mutations may yet turn out to be “secreted” Null. For example, Null_new hope_ and Null_newport_, were defined as Null on the basis of IEF and protein quantification in a period when molecular diagnosis was not widely available. A precedent for incorrect Null alleles does exist, and includes the well known M_heerlen_, which was originally classified as PiQ0 on the basis of IEF and protein quantification [44], and P_lowell_, previously called Q0_cardiff_ [45].

We describe here eight novel Null mutations in the coding regions of the *SERPINA1* gene. Three (Q0_brescia_, Q0_torino_ and Q0_cosenza_) are nonsense mutations, the others (Q0_cork_, Q0_perugia_, Q0_pordenone_, Q0_lampedusa_ and Q0_dublin_) are frameshift mutations caused by deletion of one or two nucleotides.

It is worth noting most of the new mutations reported in this study occur close to other mutations, supporting the concept of mutational hot spots in the *SERPINA1* gene [40]. In fact, Q0_brescia_ occurs in a portion of 27 nucleotides (nine amino acids) in exon III of the gene, where it is possible to find a conspicuous number of other mutations: P_lowell_ /P_duarte_/Y_barcelona_ at codon 256, Q0_cairo_ and M_pisa_ [46] at codon 259, T/S at codon 264, and the normal variant L_frankfurt_ at codon 255. Q0_pordenone_ lies in another region of 27 nucleotides together with other Null (Q0_hongkong_, Q0_new hope_) and normal (P_lyon_, P_saltlake_,) mutations. Q0_lampedusa_ occurs in the region of 21 nucleotides where, in addition to Z, other deficient (King, W_bethesda_) and normal (E_tokyo_, P_st.albans_) mutations lie. Lastly, Q0_dublin_ is only one nucleotide from M_heerlen_ and M_wurzburg_ mutations and two nucleotides from E_taurisano_ [46] deficient alleles.

While Null mutations are extremely rare, the recurrence of Q0_pordenone_ and Q0_brescia_ in certain localized areas, without evidence of consanguinity, may indicate a relatively high prevalence of each Null allele in these geographic regions.

Although a discussion of the clinical characteristics of the Null-bearing subjects presented herein is not the main purpose of this study, we can draw some interesting conclusions. Subjects with Null mutations should be considered a subgroup at particularly high risk of emphysema within the spectrum of AATD [19]. In support of this, we report three probands homozygous for Null alleles, with early onset lung disease, despite absent or modest smoking history. Interestingly, the clinical importance of Null heterozygosity has never been investigated. Here we report evidence of the recurrence of lung symptoms (dyspnoea, cough) and lung diseases (emphysema, asthma, chronic bronchitis) in M/Null subjects, over 45 years of age, irrespective of their smoking habit (Table 2).

## Conclusions

Our study has significantly expanded the list of Null alleles known to occur within the *SERPINA1* gene and underlined the importance of the correct diagnosis of this group of mutations, because of the particularly high risk of lung disease.

## Additional file

Additional file 1:
**Family trees of probands analysed in the present study.**

## Abbreviations

AAT: Alpha1-antitrypsin
SERPIN: Serine protease inhibitor
AATD: Alpha1-antitrypsin deficiency
COPD: Chronic obstructive pulmonary disease
IEF: Isoelectric focusing
HRCT: High resolution computed tomography
