# Supplementary material for: Identification and characterisation of eight novel SERPINA1 Null mutations
Source: Orphanet J Rare Dis. 2014 Nov 26;9:172. doi: 10.1186/s13023-014-0172-y (PMC4255440; doi:10.1186/s13023-014-0172-y)
Supplement: Additional file 1: — Family trees of probands analysed in the present study. [file 13023_2014_172_MOESM1_ESM.pptx]

## Slide 1
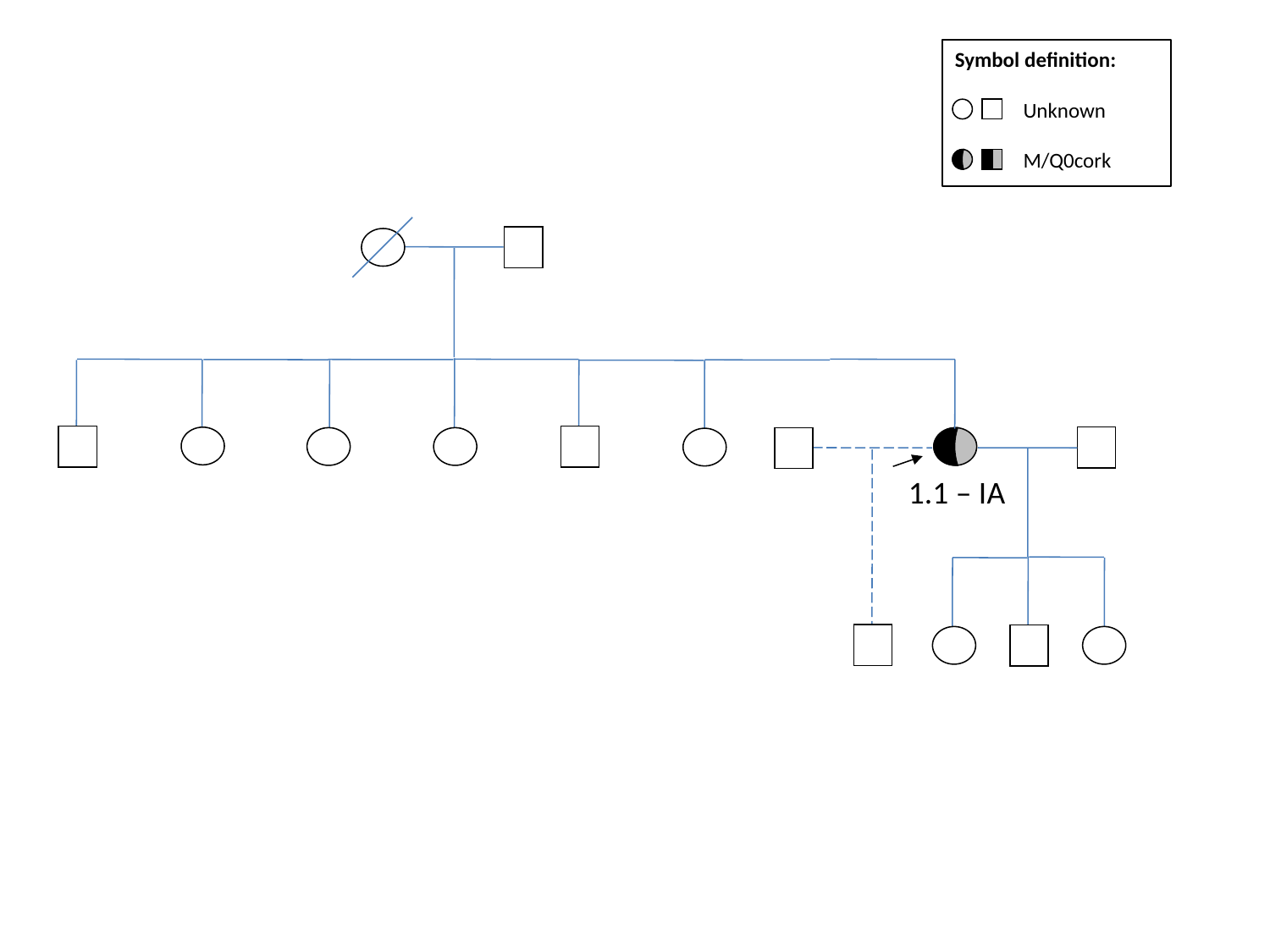

Symbol definition:
 Unknown
 M/Q0cork
1.1 – IA

## Slide 2
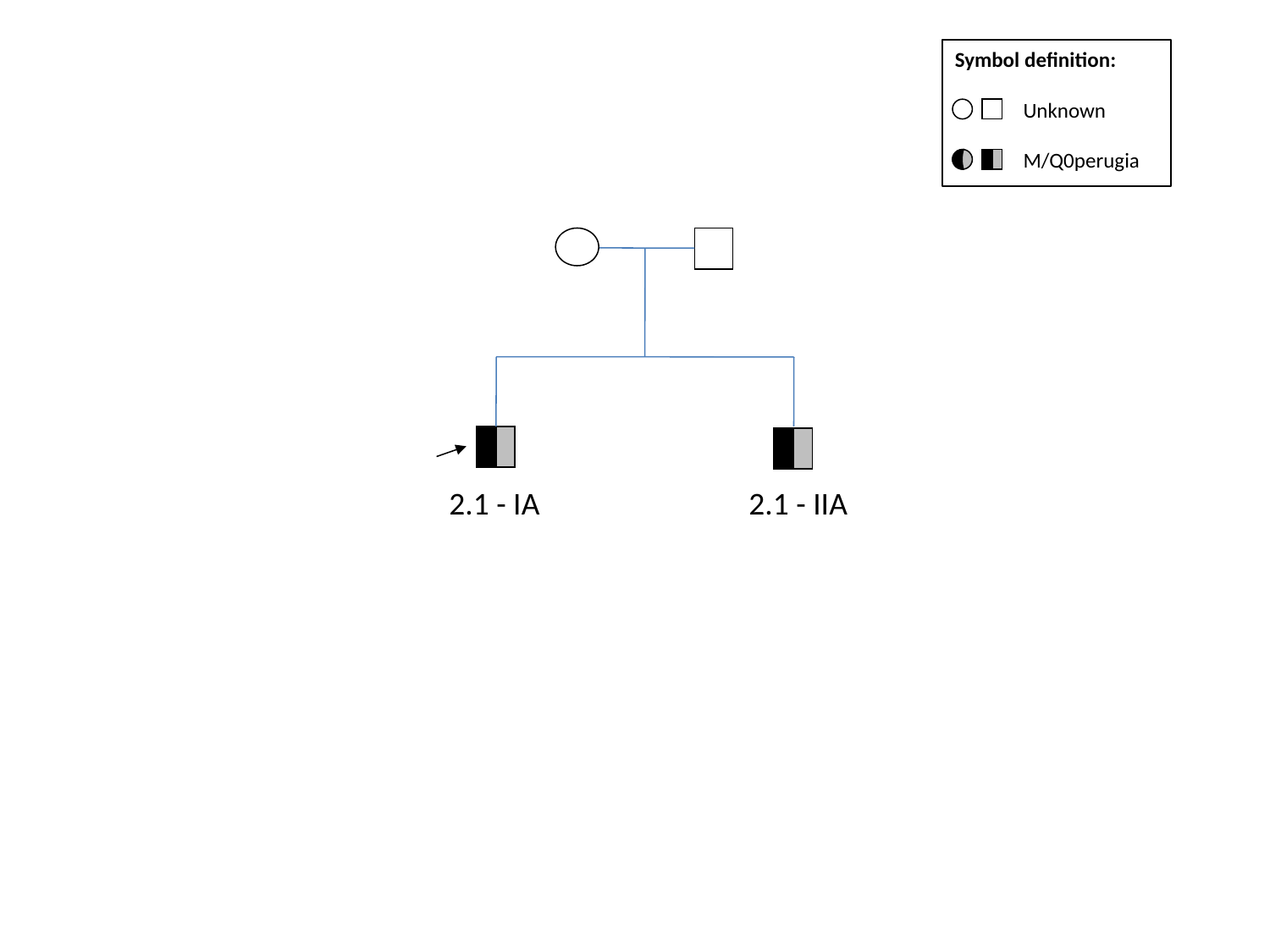

Symbol definition:
 Unknown
 M/Q0perugia
2.1 - IA
2.1 - IIA

## Slide 3
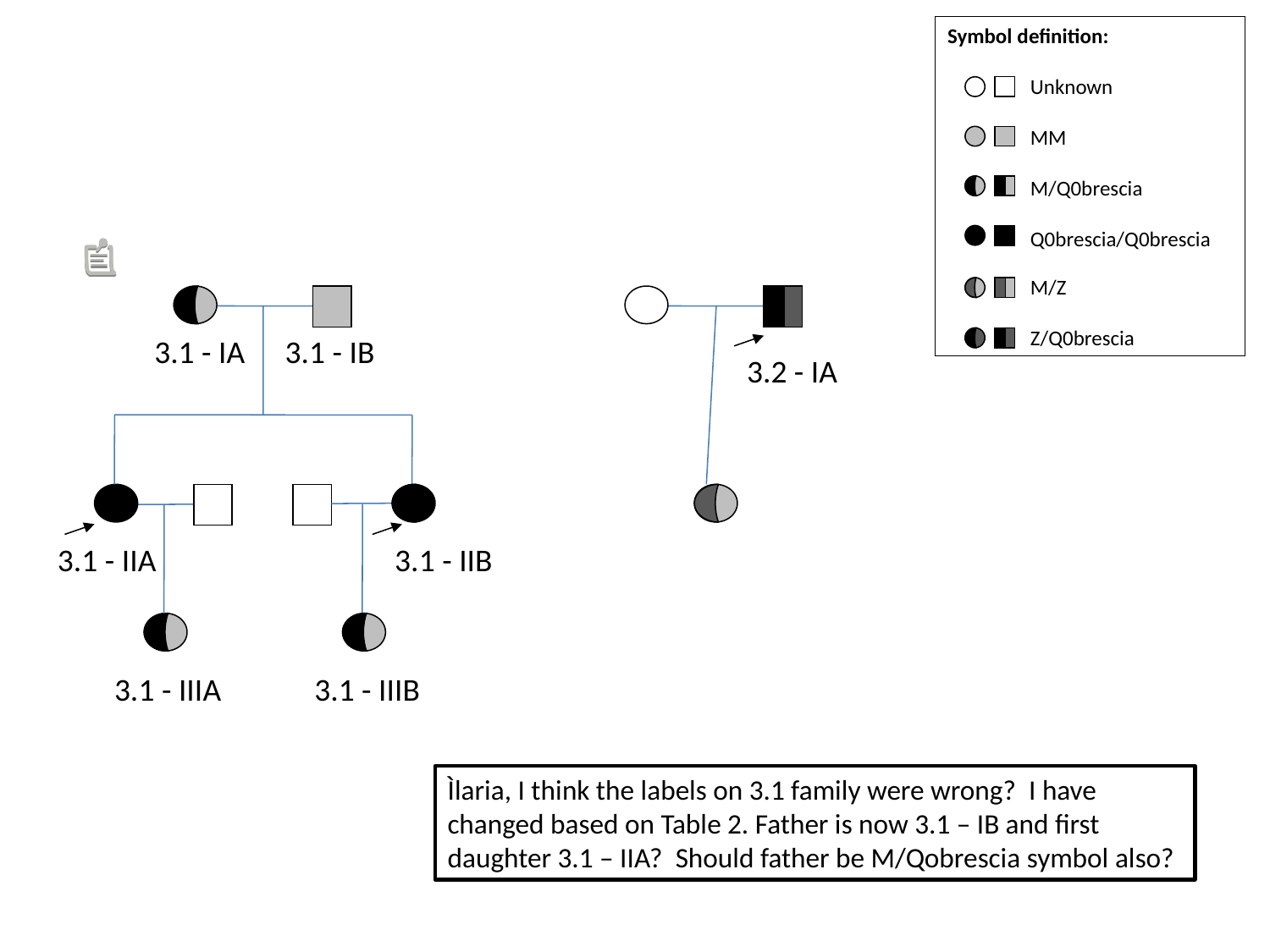

Symbol definition:
 Unknown
 MM
 M/Q0brescia
 Q0brescia/Q0brescia
 M/Z
 Z/Q0brescia
3.2 - IA
3.1 - IA
3.1 - IB
3.1 - IIA
3.1 - IIB
3.1 - IIIA
3.1 - IIIB
Ìlaria, I think the labels on 3.1 family were wrong? I have changed based on Table 2. Father is now 3.1 – IB and first daughter 3.1 – IIA? Should father be M/Qobrescia symbol also?

## Slide 4
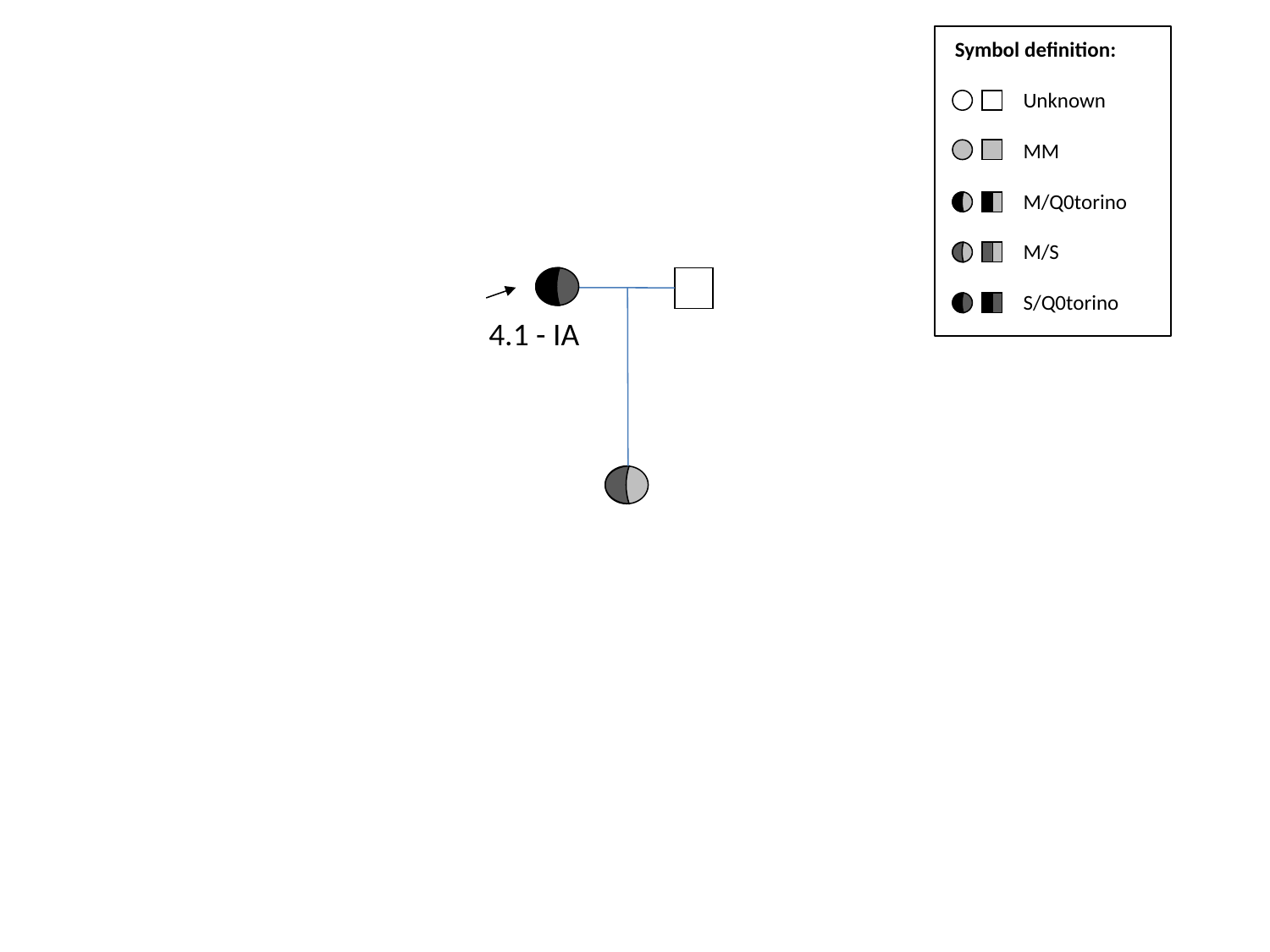

Symbol definition:
 Unknown
 MM
 M/Q0torino
 M/S
 S/Q0torino
4.1 - IA

## Slide 5
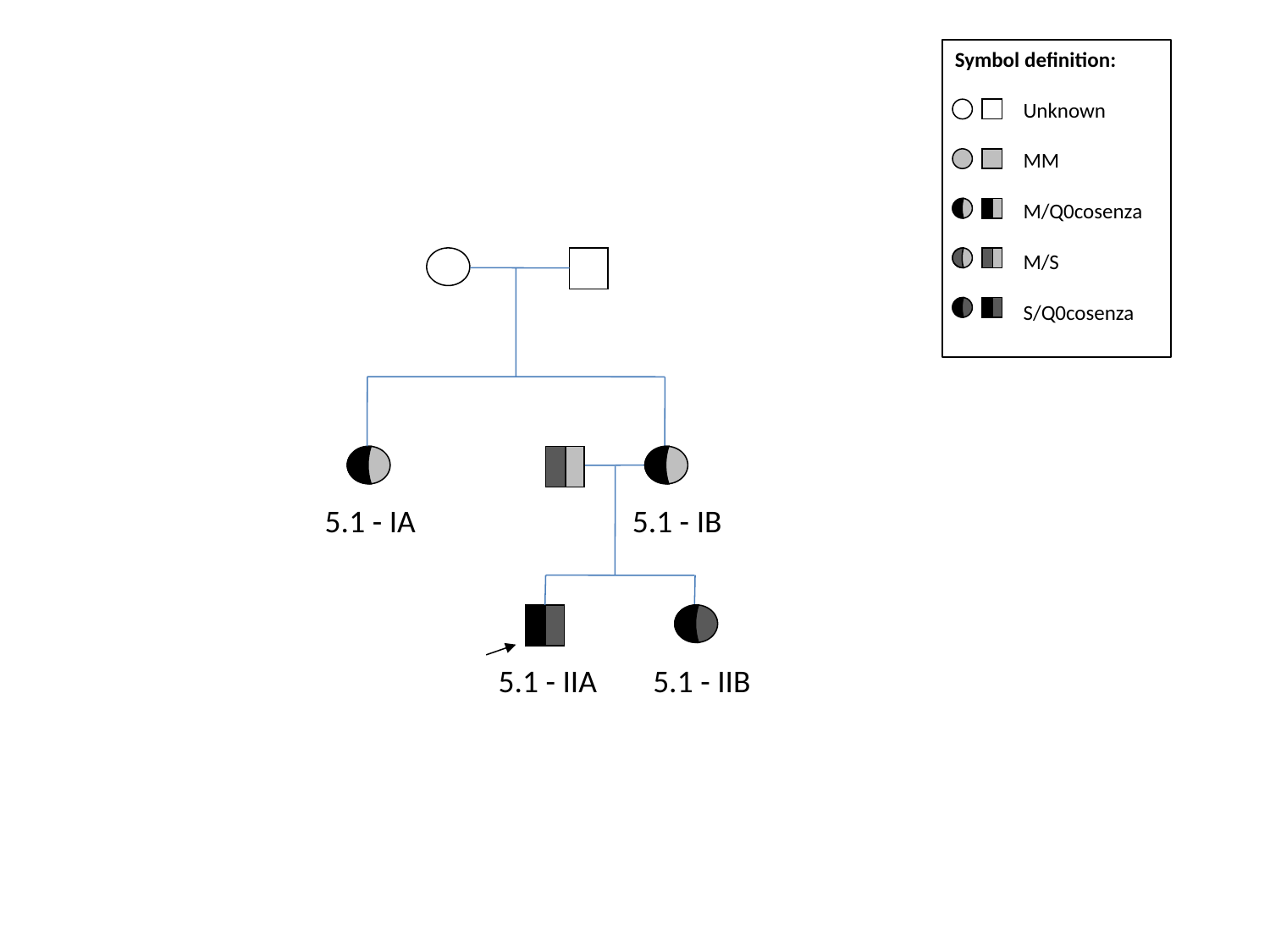

Symbol definition:
 Unknown
 MM
 M/Q0cosenza
 M/S
 S/Q0cosenza
5.1 - IA
5.1 - IB
5.1 - IIA
5.1 - IIB

## Slide 6
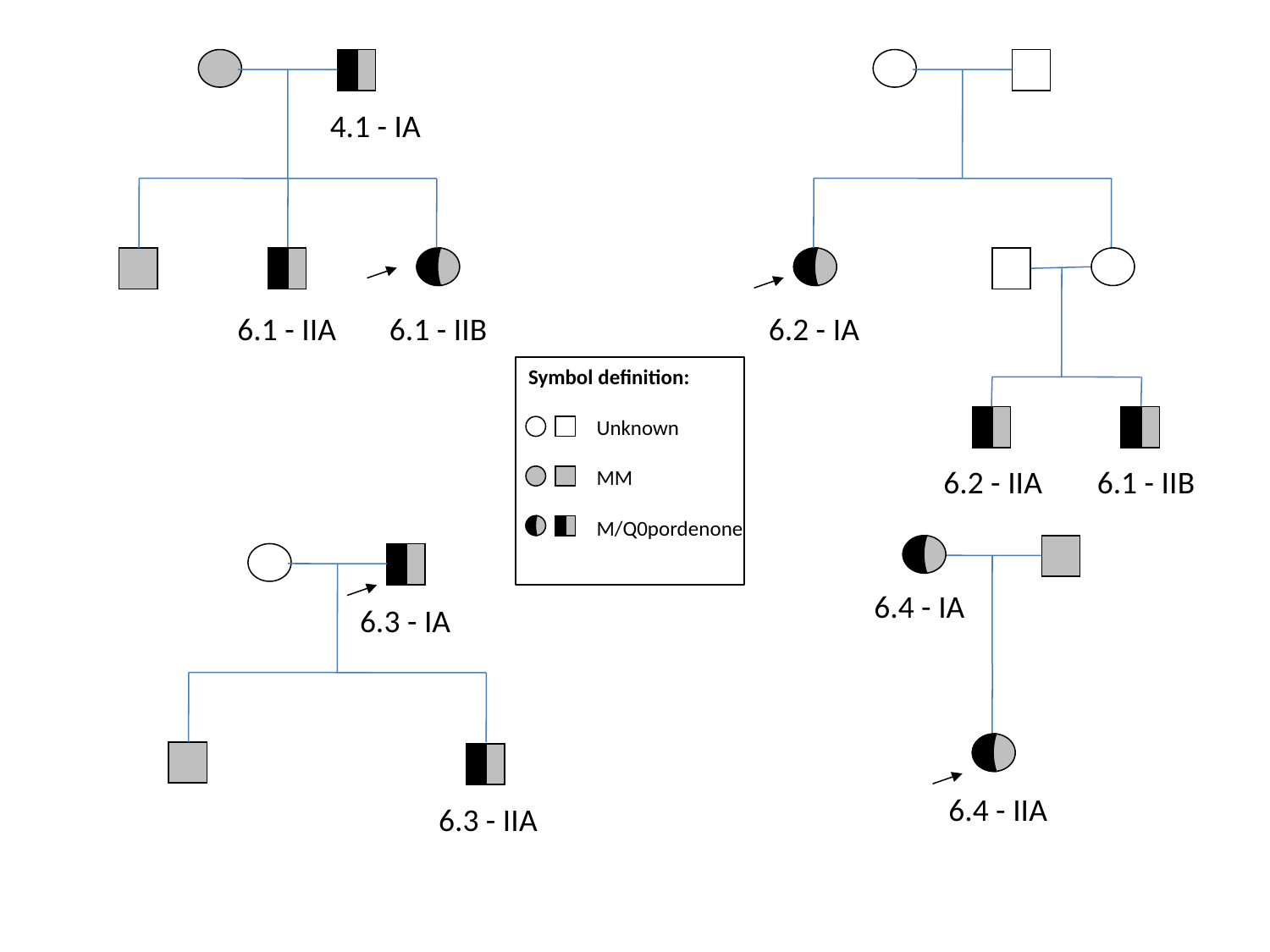

4.1 - IA
6.1 - IIA
6.1 - IIB
6.2 - IA
Symbol definition:
 Unknown
 MM
 M/Q0pordenone
6.2 - IIA
6.1 - IIB
6.4 - IA
6.4 - IIA
6.3 - IA
6.3 - IIA

## Slide 7
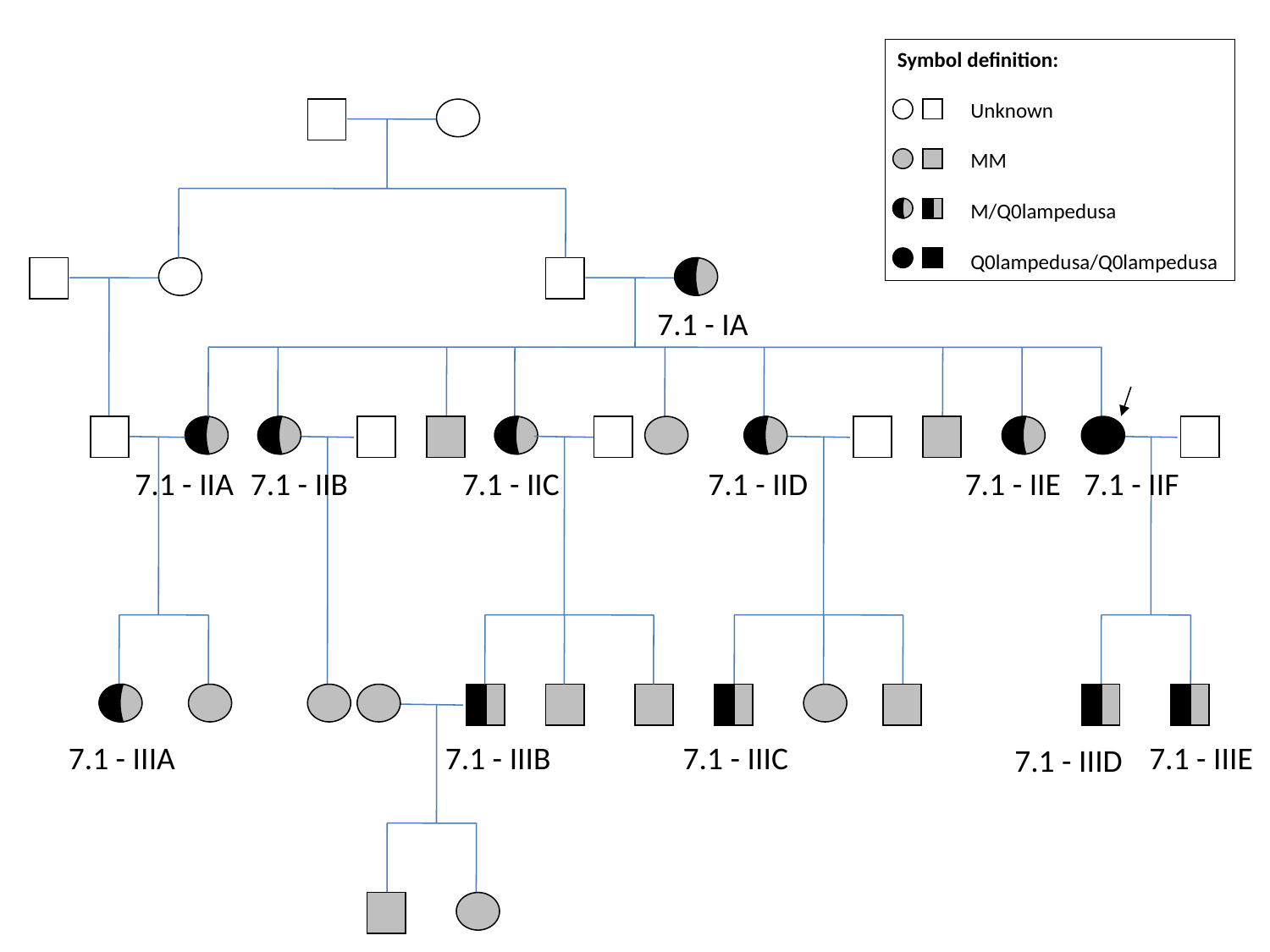

Symbol definition:
 Unknown
 MM
 M/Q0lampedusa
 Q0lampedusa/Q0lampedusa
7.1 - IA
7.1 - IIID
7.1 - IIA
7.1 - IIB
7.1 - IIC
7.1 - IID
7.1 - IIE
7.1 - IIF
7.1 - IIIA
7.1 - IIIB
7.1 - IIIC
7.1 - IIIE

## Slide 8
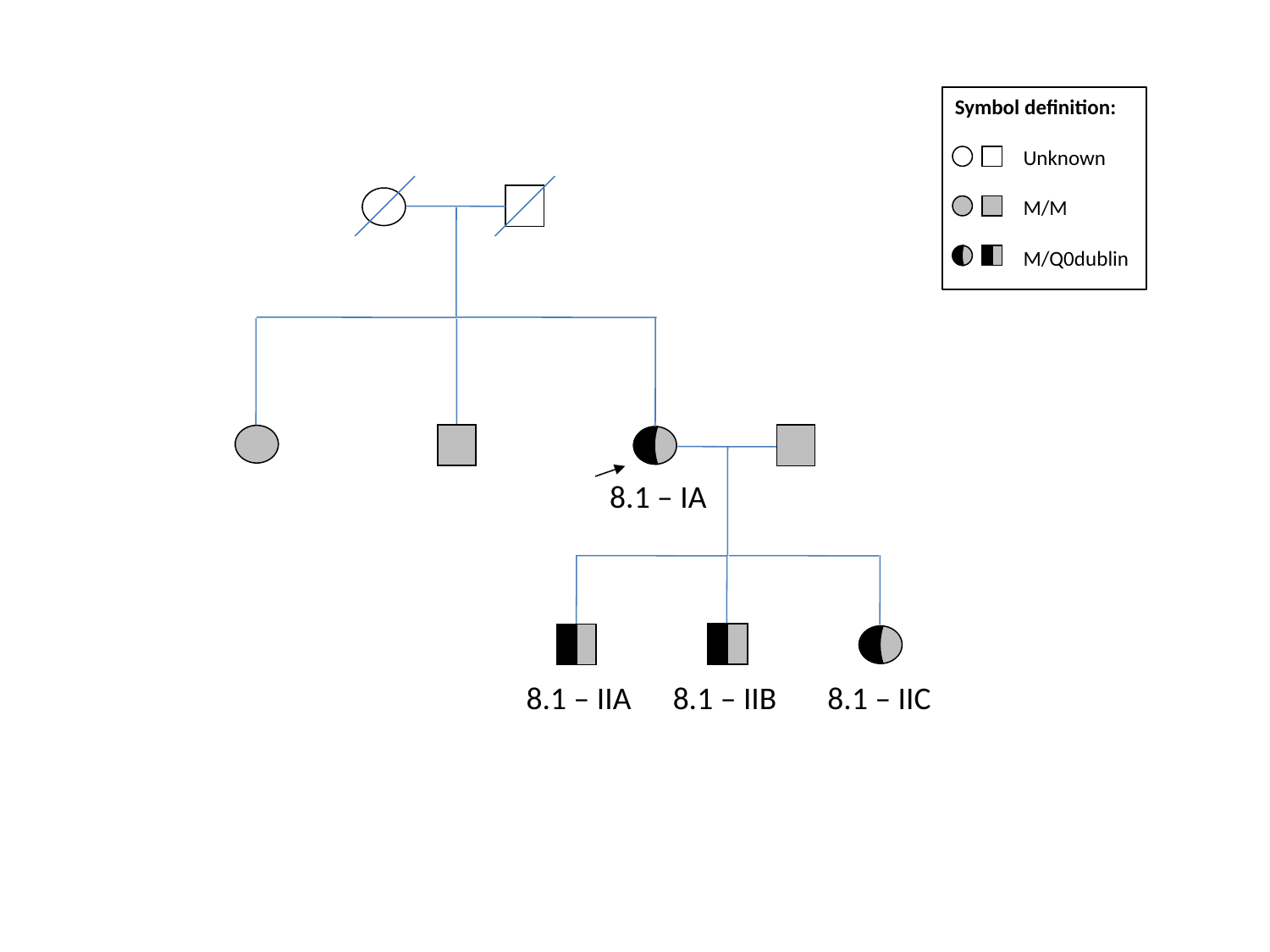

Symbol definition:
 Unknown
 M/M
 M/Q0dublin
8.1 – IA
8.1 – IIA
8.1 – IIB
8.1 – IIC
